# PEDro or Cochrane to Assess the Quality of Clinical Trials? A Meta-Epidemiological Study

**DOI:** 10.1371/journal.pone.0132634

**Published:** 2015-07-10

**Authors:** Susan Armijo-Olivo, Bruno R. da Costa, Greta G. Cummings, Christine Ha, Jorge Fuentes, Humam Saltaji, Matthias Egger

**Affiliations:** 1 CLEAR (Connecting Leadership, Education, and Research) Outcomes Research Program, University of Alberta, Faculty of Nursing, University of Alberta, Edmonton, Canada; 2 Universitat Bern, Institute of Primary Health Care, Gesellschaftstrasse 49, Bern, 3013, Switzerland; 3 Rehabilitation Research Center, Faculty of Rehabilitation Medicine, University of Alberta, Edmonton, Canada; 4 Catholic University of Maule, Department of Physical Therapy, Talca, Chile; 5 Orthodontic Graduate Program, School of Dentistry, University of Alberta, Edmonton, Alberta, Canada; 6 Institute of Social & Preventive Medicine (ISPM), University of Bern, Bern, Switzerland; Cardiff University, UNITED KINGDOM

## Abstract

**Objective:**

There is debate on how the methodological quality of clinical trials should be assessed. We compared trials of physical therapy (PT) judged to be of adequate quality based on summary scores from the Physiotherapy Evidence Database (PEDro) scale with trials judged to be of adequate quality by Cochrane Risk of Bias criteria.

**Design:**

Meta-epidemiological study within Cochrane Database of Systematic Reviews.

**Methods:**

Meta-analyses of PT trials were identified in the Cochrane Database of Systematic Reviews. For each trial PeDro and Cochrane assessments were extracted from the PeDro and Cochrane databases. Adequate quality was defined as adequate generation of random sequence, concealment of allocation, and blinding of outcome assessors (Cochrane criteria) or as trials with a PEDro summary score ≥5 or ≥6 points. We combined trials of adequate quality using random-effects meta-analysis.

**Results:**

Forty-one Cochrane reviews and 353 PT trials were included. All meta-analyses included trials with PEDro scores ≥5, 37 (90.2%) included trials with PEDro scores ≥6 and only 22 (53.7%) meta-analyses included trials of adequate quality according to the Cochrane criteria. Agreement between PeDro and Cochrane was poor for PeDro scores of ≥5 points (kappa = 0.12; 95% CI 0.07 to 0.16) and slight for ≥6 points (kappa 0.24; 95% CI 0.16-0.32). When combining effect sizes of trials deemed to be of adequate quality according to PEDro or Cochrane criteria, we found that a substantial difference in the combined effect size (≥0.15) was evident in 9 (22%) out of the 41 meta-analyses for PEDro cutoff ≥5 and 10 (24%) for cutoff ≥6.

**Conclusions:**

The PeDro and Cochrane approaches lead to different sets of trials of adequate quality, and different combined treatment estimates from meta-analyses of these trials. A consistent approach to assessing RoB in trials of physical therapy should be adopted.

## Introduction

Randomized controlled trials (RCTs) are the design of choice when comparing two or more healthcare interventions. Appropriately conducted RCTs minimize confounding and bias and thus allow causal inferences regarding the effects of interventions. However, when not appropriately done, RCTs may yield biased estimates [1–6]. Thus, it is imperative to consider the risk of bias (RoB) in RCTs when reviewing evidence for clinical decision making.

The importance of incorporating RoB assessments in evidence synthesis is widely recognized. It is good practice to ascertain whether or not results differ between trials at greater or lesser RoB. However, the approaches to perform such assessments have been inconsistent: a wide variety of checklists and scales have been developed to evaluate RoB in RCTs [7–9]. The use of different items varies between tools, some items are used without empirical evidence or theoretical rationale, and different checklists and scales are used in different research areas, suggesting lack of agreement regarding their relevance [7].

The use of summary scores from quality scales, where a study typically receives one point for each item met by the study has been criticized on several grounds [10, 11]. The effects of essential criteria, such as concealment of allocation, may be diluted or confounded by the summary quality score, if the latter includes items not related to RoB, or not important in a given context. Indeed, items that are important in some situations may not be relevant in other situations, yet they receive the same weight in the quality scale [10, 11]. For example, blinding of study participants is crucial for pain assessment or management, but irrelevant for all-cause mortality [12]. Therefore, the Cochrane Bias Methods Group and Statistical Methods Group recommend that summary scores obtained from quality scales should not be used [13]. Rather, relevant biases should be assessed one by one, including the domains of selection bias, performance bias, detection bias, attrition bias, reporting bias and other context-specific biases [14].

The debate on how best to assess the risk of bias of RCTs included in meta-analytic research has resurfaced recently in the field of physical therapy, where the Physiotherapy Evidence Database (PEDro) scale is widely used [12, 15]. Ten items (see S1 Table) contribute to a summary score, where a score of 5 or 6 typically defines adequate trial quality [12, 16–18]. Most items relate to design biases but others concern trial reporting, for example whether or not confidence intervals or other measures of variability were included in the article.

We performed a meta-epidemiological study of Cochrane systematic reviews and meta-analyses in physical therapy. Our aim was to determine the agreement between the Cochrane and the PeDro approaches to identifying physiotherapy trials of adequate quality and to examine whether or not the approach chosen (PEDro or Cochrane) may affect the conclusions of meta-analyses in physical therapy.

## Methods

### Literature search and eligibility criteria

We searched the Cochrane Database of Systematic Reviews (CDSR) from Jan 1 2005 to May 25 2011 for meta-analyses of physical therapy interventions using the free-text words ‘physical therapy’, ‘physiotherapy’, ‘rehabilitation’, ‘exercise’, ‘electrophysical agents’, ‘acupuncture’, ‘massage’, ‘transcutaneous electrical stimulation (TENS)’, ‘interferential current’, ‘ultrasound’, ‘stretching’, ‘chest therapy’, ‘pulmonary rehabilitation’, ‘manipulative therapy’, ‘mobilization’, and related terms. For the detailed search strategy see S1 Appendix. Meta-analyses were eligible if they included at least three RCTs of physical therapy interventions according to the World Confederation for Physical Therapy (WCPT) [19] with a continuous outcome. If there were several eligible outcomes, we chose the primary outcome as specified by the authors. If the primary outcome was not eligible or not specified, the outcome that contained the largest number of trials was chosen.

### PEDro Scores and Cochrane RoB assessment

When available, quality assessments of RCTs included in reviews were obtained from the PEDro database [16] (see also http://www.pedro.org.au) or the Cochrane reviews. If a trial was not included in the PEDro database or no Cochrane RoB assessment had been done, we performed the assessments ourselves. Two reviewers (CH, DP, AC, JF, or HS) independently assessed trials, with discrepancies resolved by discussion or consultation with S.A-O. We trained assessors using 10 trials not included in the study, based on relevant guidelines [13, 14, 20]. As described in detail elsewhere, the PEDro and Cochrane training assessments were discussed in a group meeting to determine consistency in ratings, and calibrate assessments [21]. We defined trials of adequate quality as having adequate generation of random sequence, concealment of allocation, and blinding of outcome assessors (based on the Cochrane RoB tool) or as trials with a PEDro summary score of at least 5 or 6 points, the cutoffs widely used in the literature [12, 16–18].

### Data extraction of treatment estimates and trial characteristics

Two reviewers independently extracted data on means, standard deviations, standard errors, and sample sizes from each RCT. Data on the design of the trial, type of intervention (including information on intensity, frequency, dosage), condition, outcome (objective, subjective), funding source, publication year, and statistical analysis were also collected. We defined outcomes as objective or subjective following the approach by Wood et al [5].

### Statistical analysis

We calculated the kappa (κ) statistics for categorical data to assess the agreement between the PeDro scores and the Cochrane approach for classifying trial quality. We used the criteria proposed by Byrt to interpret kappa values [22]: values of 0.93 to 1 represent excellent agreement; 0.81 to 0.92 very good agreement; 0.61 to 0.80 good agreement; 0.41 to 0.60 fair agreement; 0.21 to 0.40 slight agreement, 0.01 to 0.20 poor agreement; and less than 0.01 no agreement.

We calculated standardized effect sizes for each trial using Cohen’s approach [23] using approximations when necessary [24]. We followed the Cochrane reviews to determine the comparison included for analysis (i.e. treatment of interest and control group). The statistical analysis allowed both for heterogeneity between trials within a meta-analysis and for heterogeneity between meta-analyses.[25] In a first step we used inverse-variance random-effects meta-analyses to combine effect sizes across trials and calculated the DerSimonian and Laird estimate of the between trial variance (tau squared).[26] Calculations were done separately for trials classified as of adequate quality based on PEDro summary scores and for trials of adequate quality according to the Cochrane RoB tool. We combined effect sizes from trials of adequate quality according to PeDro scores or Cochrane approach for each meta-analysis.

Differences in combined estimates between PeDro and Cochrane were considered relevant if they corresponded to 0.15 standard deviation units or more, a difference that corresponds to a clinically relevant treatment effect [27–30].

Stata statistical software (version 12, College Station, Texas) was used to perform the analyses. Results are presented as kappa statistics or standardized effect sizes with 95% confidence intervals (CI). The study was approved by the Ethics Board of the University of Alberta (Pro00038172).

## Results

### Selection and characteristics of meta-analyses and randomised trials

**Fig 1:**
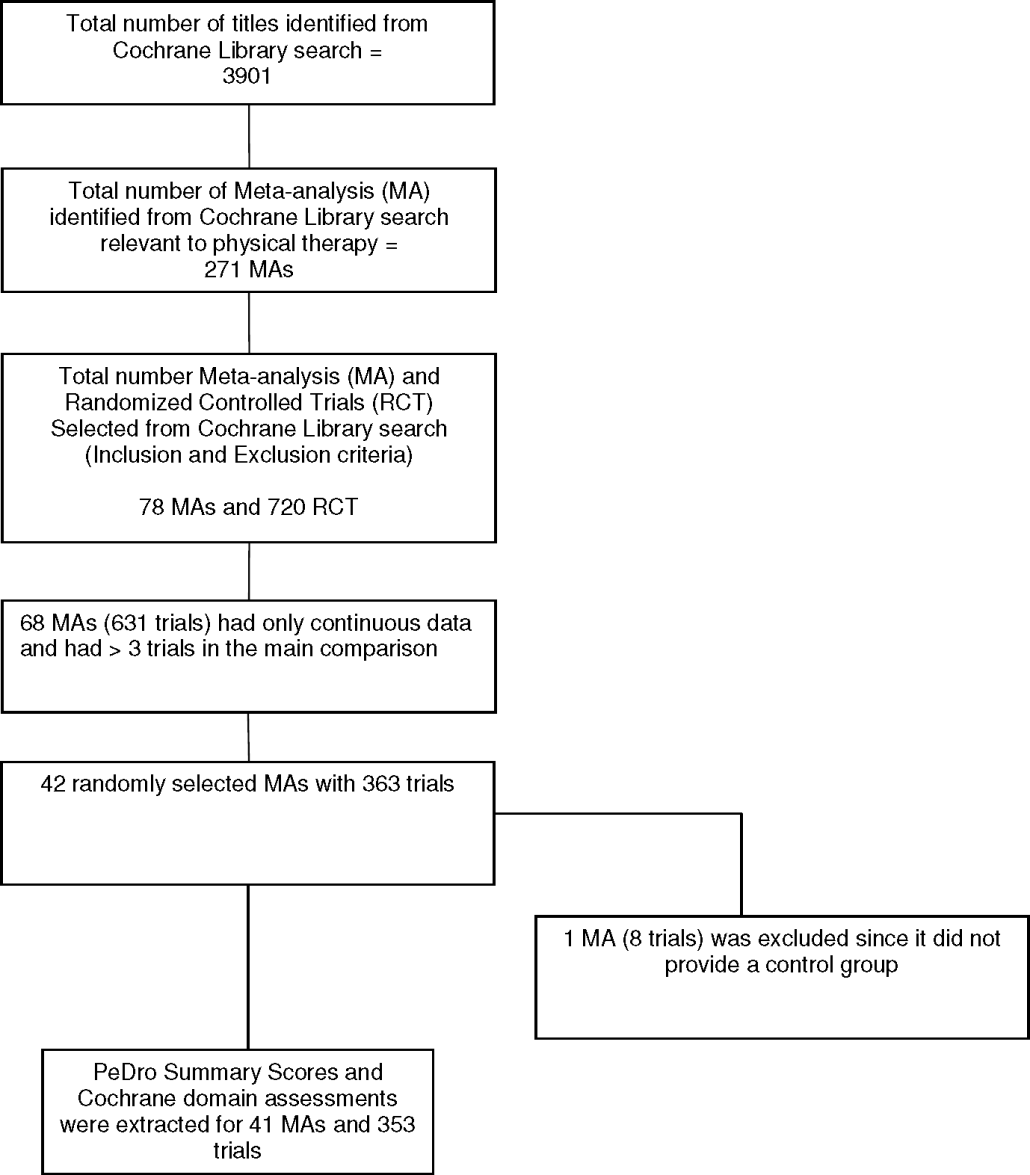
Diagram for identification of studies.

The search identified 3901 Cochrane reviews, with 271 reviews potentially relevant to physical therapy. Of these, 68 reviews included a meta-analysis of at least three studies of physical therapy interventions and used a continuous outcome. We randomly selected 42 meta-analyses but excluded one [31] because it used follow-up data from the same group rather than a control group for comparison (Fig 1). Forty-one meta-analyses, 353 trials and 42,342 patients contributed to the analysis. Table 1 and S2 Table detail the characteristics of the reviews. Briefly, the reviews were published between 2008 and 2011 and included meta-analyses of the effectiveness of physical therapy interventions for musculoskeletal (22 reviews) [32–40] cardiorespiratory (8 reviews) [41-48], neurological (6 reviews) [49–55], and other areas of physical therapy (5 reviews) [55–59]. A median number of 6 trials were included in each meta-analysis (interquartile range 5–8). Most trials were parallel group trials (330; 93%), single-center studies (270; 76.5%) and had active control interventions (325; 91.5%). Trials compared two groups (222; 62.9%), three groups (82; 23.2%) or four or more groups (49; 13.9%). The most common intervention was exercise (n = 246, 69.7%). Electrophysical agents, manual therapy, education, and acupuncture were used in 15 (4.2%), 14 (4.0%), 10 (2.8%), and 8 trials (2.3%) respectively. The remaining trials used a combination of exercise and physical agents, manual therapy and other treatments such as respiratory exercises.

**Table 1:** Meta-analysis and trial characteristics.

Meta-analyses	Musculoskeletal	Cardio-respiratory	Neurology	Other	Total
Total No. of meta-analyses	22	8	6	5	41
Median No. of included trials (range)	6 (3–33)	7.5 (5–15)	6.5 (5–23)	6 (6–17)	6 (3–33)
Median No. of participants (range)	363 (122–3616)	1079 (201–3109)	282.5 (91–907)	556 (236–7598)	379 (91–7598)
Total No. of patients included	19861	8397	2138	11946	42,342
***Main intervention***					
Exercise	13	6	3	4	30
Physical agents	1	0	1	0	2
Acupuncture	2	0	0	0	2
Manual therapy	1	0	0	0	1
Other	1	2	2	1	6
***Outcomes***					
Clinician assessed outcome	8	4	6	3	21
Self-reported outcome	11	3	0	1	15
Administrative data/automated outcome/laboratory	3	1	0	1	5
**Trials**					
Total No. of trials	192	67	52	42	353
Parallel group trial	190	62	47	40	339
Single center trial	150	49	43	32	274
Active control interventions	90	27	22	12	151

### Trials of adequate quality according to PeDro scores and Cochrane RoB tool

PEDro scores were obtained from the PEDro database for 333 trials (94.3%) and determined by us for 20 trials (5.7%). Similarly, Cochrane RoB assessments were available from the Cochrane reviews for 314 trials (89.0%) and done by us for 39 (11.0%) trials. A total of 97 (27.5%), 70 (19.8%), 50 (14.2%) and 36 (10.2%) trials had PEDro summary scores of 5, 6, 7, or 8 points, respectively. Among trials with PEDro summary scores of 5 (97 trials), only 11 trials (11.3%) were of adequate quality according to the Cochrane RoB domain approach. The corresponding numbers for 6, 7 or 8 points on the PEDro scale were 9 trials (12.9%), 14 trials (28%) and 20 trials (55.6%) (Table 2). Only few trials of adequate quality based on the PeDro scale had adequate allocation concealment or blinding of outcome assessors. For example, among the 97 trials with a PEDro score of 5 points, only 21 (21.6%) had adequate concealment of allocation and 23 trials (23.7%) had adequate blinding of assessors (Table 2).

**Table 2:** Distribution of 353 trials across PEDro scores and number of trials and percentage classified as of adequate quality according to the Cochrane RoB tool.

PEDro Score	Total No. of trials (Column %)	No. of adequate quality trials (row %)	No. of trials with adequate concealment of allocation (row %)	No. of trials with adequate blinding of outcome assessors (row %)
1	3 (0.8)	0 (0)	0 (0)	0 (0)
2	7 (2)	0 (0)	0 (0)	2 (28.6)
3	33 (9.3)	2 (5.7)	4 (12.1)	5 (15.1)
4	53 (15)	1 (1.9)	1 (1.9)	8 (15.4)
5	97 (27.5)	11 (11.3)	21 (21.6)	23 (23.7)
6	70 (19.8)	9 (12.9)	26 (37.1)	30 (42.9)
7	50 (14.2)	14 (28)	30(60)	23 (46)
8	36 (10.2)	20 (55.6)	29 (80.6)	24 (66.7)
9	4 (1.1)	3 (75.0)	4 (100)	43(75)
10	0 (0)	0 (0)	0 (0)	0 (0)

### Agreement on adequate quality between PeDro scores and Cochrane RoB tool

**Fig 2:**
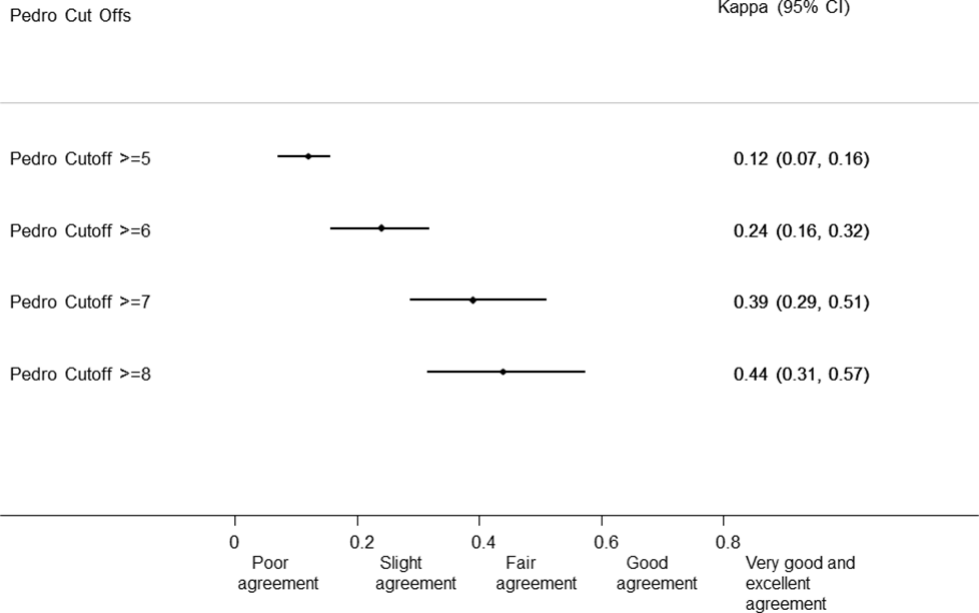
Agreement between PeDro Score at different cut offs and Cochrane Approach.

Agreement between PeDro and Cochrane for the definition of adequate quality across all meta-analyses was poor for PeDro scores >5 or more (kappa 0.12; 95% CI 0.07–0.16), slight for a score >6 or more (kappa 0.24; 95% CI 0.16–0.32), and 7 or more (kappa 0.39; 95% CI 0.286–0.510), and fair (kappa 0.44; 95% CI 0.314–0.574) for 8 points and more (Fig 2).

### Differences in treatment effects between trials of adequate quality trials defined according to PEDro scores and Cochrane RoB tool

All 41 meta-analyses included adequate quality trials based on a PEDro score of 5 or more, and 37 (90.2%), 30 (73.2%) and 19 (46.3%) meta-analyses included adequate quality trials based on scores of at least 6, 7 or 8. In contrast, 22 (53.7) meta-analyses did not include any adequate quality trials using the Cochrane RoB domain approach (S3 Table). An extreme example was the meta-analysis by Liu et al. [60] which included only one trial of adequate quality according to the Cochrane approach but 29, 18, 9, and 5 trials of adequate quality when using PeDro scores of 5, 6, 7, and 8 points, respectively.

When combining effect sizes of trials deemed to be of adequate quality according to PEDro or Cochrane criteria, we found that a substantial difference in the combined effect size (> 0.15) was evident in 9 (22%) out of the 41 meta-analyses for PEDro cutoff >5 and 10 (24%) for the cutoff >6 (Table 3). In addition to this difference, 19 and 15 systematic reviews (46% and 37%) did not have adequate quality trials by Cochrane approach but they had trials of adequate quality by PeDro ≥5 points and ≥6 points respectively. Considering this as a discrepancy as well, an overall discrepancy existed between 2 approaches in 28 meta-analyses (68%) and 25 meta-analyses (61%) for PeDro ≥5 points and ≥6 points respectively.

**Table 3:** Combined effect sizes from trials of adequate quality using the PEDro or Cochrane approach to assess trials, and differences in effect sizes between results obtained with Cochrane and PEDro.

Meta-analysis	Pedro Cutoff ≥5		Pedro Cutoff ≥6		Cochrane Adequate Quality		Difference Pedro-Cochrane	
	No. of trials	Combined effect size (95%CI)	No. of trials	Combined effect size (95%CI)	No. of trials	Combined effect size (95%CI)	Pedro Cutoff >5	Pedro Cutoff >6
Pollock A, 2009	4	-0.28 (-0.59, 0.03)	4	-0.28 (-0.59, 0.03)	1	-0.34 (-0.66, -0.03)	0.06	0.06
States R, 2009	6	-0.44 (-0.89, 0.00)	5	-0.51 (-0.99, -0.03)	3	-0.34 (-0.71, 0.03)	-0.1	-0.17*
Schaafsma F, 2011	5	-0.18 (-0.37, -0.00)	2	-0.23 (-0.57, 0.12)	3	-0.10 (-0.32, 0.11)	-0.08	-0.13
Markes M, 2009	2	-0.46 (-1.02, 0.11)	0	NAQT	0	NAQT	-	-
McNeely M, 2010	3	-0.33 (-1.41, 0.75)	2	0.03 (-1.97, 2.03)	0	NAQT	-	-
Main E, 2010	1	8.26 (0.75, 15.77)	1	8.26 (0.75, 15.77)	0	NAQT	-	-
Davies E, 2010	8	-0.48 (-0.74, -0.23)	5	-0.35 (-0.63, -0.07)	1	-0.01 (-0.36, 0.35)	-0.47*	-0.34*
Busch A, 2008	5	-0.24 (-0.66, 0.18)	4	-0.28 (-0.85, 0.29)	1	-0.15 (-0.58, 0.28)	-0.09	-0.13
Liu C, 2009	29	-0.14 (-0.24, -0.04)	18	-0.15 (-0.27, -0.04)	1	-0.09 (-0.72, 0.55)	-0.05	-0.06
Furlan A, 2011	2	0.21 (-1.12, 1.54)	2	0.21 (-1.12, 1.54)	1	0.29 (-0.16, 0.74)	-0.08	-0.08
Fransen M, 2009	5	-0.34 (-0.85, 0.17)	5	-0.34 (-0.85, 0.17)	4	-0.45 (-1.03, 0.14)	0.11	0.11
Ostelo R, 2011	2	-1.11 (-2.13, -0.09)	2	-1.11 (-2.13, -0.09)	0	NAQT	-	-
Taylor R, 2010	6	0.09 (-0.16, 0.34)	3	0.18 (-0.43, 0.79)	4	-0.12 (-0.39, 0.15)	0.21*	0.3*
Harvey L, 2010	6	-0.45 (-0.69, -0.21)	3	-0.44 (-0.72, -0.16)	3	-0.28 (-0.62, 0.07)	-0.17*	-0.16*
Mead GE, 2010	13	-0.87 (-1.27, -0.47)	7	-0.65 (-1.10, -0.20)	3	-0.41 (-0.83, 0.00)	-0.46*	-0.24*
Edmonds M, 2010	5	-0.78 (-1.28, -0.28)	3	-1.12 (-1.59, -0.66)	0	NAQT	-	-
Howe TE, 2008	3	-0.18 (-0.44, 0.09)	3	-0.18 (-0.44, 0.09)	1	-0.17 (-0.72, 0.38)	-0.01	-0.01
Fransen M, 2009	28	-0.43 (-0.55, -0.32)	21	-0.36 (-0.45, -0.26)	10	-0.31 (-0.45, -0.16)	-0.12	-0.05
Lin CH, 2008	3	-0.50 (-1.06, 0.06)	1	-0.14 (-0.49, 0.21)	3	-0.41 (-0.84, 0.02)	-0.09	0.27*
Rutjes AW, 2010	5	-0.49 (-0.76, -0.23)	4	-0.43 (-0.74, -0.11)	0	NAQT	-	-
Woodford HJ, 2009	3	0.04 (-0.53, 0.61)	2	-0.02 (-0.66, 0.62)	0	NAQT	-	-
Saunders DH, 2009	5	-0.33 (-0.52, -0.13)	5	-0.33 (-0.52, -0.13)	0	NAQT	-	-
O'Brien K, 2010	3	-1.16 (-1.56, -0.76)	0	NAQT	0	NAQT	-	-
Sirtoti V, 2009	6	-0.37 (-0.68, -0.07)	5	-0.36 (-0.72, 0.00)	2	-0.40 (-1.12, 0.32)	0.03	0.04
Hayden J, 2011	13	-0.21 (-0.31, -0.11)	8	-0.19 (-0.30, -0.07)	0	NAQT	-	-
Orozco LJ, 2008	6	-0.22 (-0.42, -0.01)	5	-0.22 (-0.46, 0.02)	2	-0.46 (-0.54, -0.38)	0.24*	0.24*
De Morton N, 2009	2	-0.12 (-0.33, 0.09)	2	-0.12 (-0.33, 0.09)	0	NAQT	-	-
Mehrholz J, 2010	6	-0.53 (-0.89, -0.17)	0	NAQT	2	-0.25 (-0.90, 0.39)	-0.28*	-
Shaw K, 2009	9	-0.37 (-0.59, -0.14)	3	-0.24 (-0.51, 0.03)	0	NAQT	-	-
Handholl H, 2009	6	-0.10 (-0.37, 0.17)	5	-0.11 (-0.44, 0.22)	1	0.40 (0.16, 0.64)	-0.5*	-0.51*
Effing T, 2009	6	-0.13 (-0.28, 0.01)	3	-0.10 (-0.29, 0.09)	2	-0.16 (-0.36, 0.05)	0.03	0.06
Bendermacher B, 2009	2	-1.17 (-1.65, -0.68)	0	NAQT	0	NAQT	-	-
Bonaiuti D, 2009	4	-0.63 (-1.12, -0.14)	2	-0.62 (-1.34, 0.11)	0	NAQT	-	-
Foster C, 2009	13	-0.18 (-0.32, -0.04)	5	-0.09 (-0.25, 0.07)	0	NAQT	-	-
Jolliffe J, 2009	7	-0.67 (-1.01, -0.32)	2	-0.70 (-1.76, 0.36)	1	-1.23 (-1.50, -0.95)	0.56*	0.53*
Katalinic O, 2010	6	0.22 (-0.13, 0.56)	6	0.22 (-0.13, 0.56)	5	0.27 (-0.16, 0.71)	-0.05	-0.05
Puhan M, 2010	4	-0.70 (-1.28, -0.12)	2	-0.36 (-1.15, 0.42)	0	NAQT	-	-
Kramer M, 2010	1	-0.53 (-1.12, 0.06)	1	-0.53 (-1.12, 0.06)	0	NAQT	-	-
Rutjes AW, 2010	4	-1.55 (-2.21, -0.89)	2	-1.26 (-1.91, -0.61)	0	NAQT	-	-
Watson, 2008	5	-1.16 (-2.25, -0.06)	2	-1.05 (-3.63, 1.52)	0	NAQT	-	-
Manheimer E, 2010	7	-0.29 (-0.48, -0.10)	7	-0.29 (-0.48, -0.10)	3	-0.14 (-0.34, 0.06)	-0.15*	-0.15*

NAQT; no adequate quality trial included in meta-analysis.

* Difference clinically relevant.

## Discussion

In this meta-epidemiological study we found that depending on the approach used to assess the risk of bias, PEDro scores or Cochrane criteria, different trials were considered to be of adequate quality. Unsurprisingly the combined estimates of treatment effects from these adequate quality trials differed substantially, depending on the approach chosen and the cutoff score used to define adequate quality. This may have important implications for decision making since different recommendations will be made based on different treatment effects obtained from meta-analyses of trials considered of adequate quality.

There were substantial disagreements between the two methods regarding which and how many trials are considered to be of adequate quality. Almost 60% of trials were considered to be of adequate quality based on the PeDro cut off of ≥5 points, which is widely used in the literature [12, 16–18]. However, many of these trials did not meet the accepted quality standards such as generation of random sequence, concealment of allocation, and blinding of study assessors defined by the Cochrane RoB tool. Previous studies have shown that these trial features can have a substantial impact on the estimates of treatment effect [4, 5, 61–63]. For example, inadequate allocation concealment may overestimate treatment effects by 5% to 30% [4, 5, 64–66] and lack of double-blinding may overestimate effects by 9% to 44% [3, 5, 66]. Biased estimates from individual trials can lead to biased results and misleading conclusions in systematic reviews and meta-analyses [5, 61, 67–69]. This can in turn affect patient care through different recommendations and decisions in clinical practice. Indeed, the differences observed in our study are clinically relevant: in a substantial proportion of meta-analyses the differences in effect sizes between the two approaches was 0.15 or greater. The typical treatment effect in physical therapy is in the range of 0.1 to 0.8 [27–30].

Our results are consistent with studies [10–12] that showed that bias may be introduced when summary quality scores are used as an eligibility criterion for trials to be included in systematic reviews and meta-analyses. Analyzing a smaller number of trials, Greenland [10], Colle [70], and Juni and colleagues [11] showed that using different tools for evaluating quality of primary research in meta-analyses can lead to different results. Summary scores dilute the effect of items that are important for the risk of bias with items that are not related to the internal validity of trials, but to the quality of reporting of trials. Although transparent reporting is important to assess the quality of trial conduct, a focus on quality of reporting in quality scores can hide differences in trial conduct and lead to under- or over-estimation of the methodological quality. [71]

Interestingly, despite having been developed for clinical trials of physical therapy the PEDro scale does not contain items specific to this field. Because physical therapy clinical trials are more complex than drug trials, compliance and standardization of treatment protocols, reliable application of the intervention [72], and skills, training, and experience of therapists are all issues of particular importance to physical therapy [73].

To the best of our knowledge, this is the first meta-epidemiological study addressing the question of how best to assess trials for inclusion in meta-analyses in physical therapy. One of the main strengths of this study is the large number of meta-analyses and trials included. Most previous studies looked at one systematic review only [11, 12, 70]. We restricted our analysis to Cochrane systematic reviews in physical therapy and results might not be applicable to all Cochrane reviews conducted in other areas of research. However, similar results have been previously obtained in different areas of health research with smaller sample of trials and meta-analyses [11, 12, 70]

In conclusion, we found that the PeDro and Cochrane approaches to identifying RCTs of adequate quality lead to different sets of trials and different combined treatment estimates from meta-analyses of these trials. A consistent approach to assessing RoB in trials of physical therapy based on the Cochrane RoB tool rather than a summary score from the PEDro scale should be adopted.

## Supporting Information

S1 Appendix Search strategy to identify systematic review in physical therapy from the Cochrane Library of Systematic Reviews. (DOC)

S1 Table Items of the Physiotherapy Evidence Database (PEDro) scale. (DOCX)

S2 Table Characteristics of meta-analyses included in the study. (DOCX)

S3 Table Identity of trials of adequate quality based on different PeDro cutoffs, Cochrane approach, and results from the original Cochrane review. (DOC)

S4 Table Data for manuscript. (XLS)
