# Supplementary material for: PEDro or Cochrane to Assess the Quality of Clinical Trials? A Meta-Epidemiological Study
Source: PLoS One. 2015 Jul 10;10(7):e0132634. doi: 10.1371/journal.pone.0132634 (PMC4498768; doi:10.1371/journal.pone.0132634)
Supplement: S1 Table — (DOCX) [file pone.0132634.s002.docx]

**Table S1.** Items of the Physiotherapy Evidence Database (PEDro) scale.

| Item No. | Description |
| --- | --- |
| 1 | Eligibility criteria were specified |
| 2 | Subjects were randomly allocated to groups |
| 3 | Allocation was concealed |
| 4 | The groups were similar at baseline regarding the most important prognostic indicators |
| 5 | There was blinding of all subjects |
| 6 | There was blinding of all therapists who administered the therapy |
| 7 | There was blinding of all assessors who measured at least one key outcome |
| 8 | Measures of at least one key outcome were obtained from more than 85% of the subjects initially allocated to groups |
| 9 | All subjects for whom outcome measures were available received the treatment or control condition as allocated or, where this was not the case, data for at least one key outcome was analyzed by “intention to treat” |
| 10 | The results of between-group statistical comparisons are reported for at least one key outcome |
| 11 | The study provides both point measures and measures of variability for at least one key outcome |

Note: Item No. 1 (eligibility criteria) does not contribute to total score
