# Supplementary material for: PEDro or Cochrane to Assess the Quality of Clinical Trials? A Meta-Epidemiological Study
Source: PLoS One. 2015 Jul 10;10(7):e0132634. doi: 10.1371/journal.pone.0132634 (PMC4498768; doi:10.1371/journal.pone.0132634)
Supplement: S2 Table — (DOCX) [file pone.0132634.s003.docx]

**Table S2.** Characteristics of selected meta-analyses.

| **Author** | | **Year** | | **# of trials** | **Pt in tx group** | **Pt in control group** | **# of Pt included** | **Outcome** | **Outcome Source** | **Type of outcome** | **Area** | **Specific Area** |
| --- | --- | --- | --- | --- | --- | --- | --- | --- | --- | --- | --- | --- |
| Pollock, A | | 2009 | | 5 | 213 | 209 | 422 | Functional Independence Scale | Clinician assessment | Ob | Neuro | Other |
| States, R | | 2009 | | 6 | 195 | 186 | 381 | Gait speed (m/s) (higher is better) | Clinician assessment | Ob | Neuro | Exercise |
| Schaafsma, F | | 2011 | | 5 |  |  | 1057 | Time to return to work (lower is better) | Administrative data | Ob | MSK | Exercise |
| Markes, M | | 2009 | | 4 | 127 | 80 | 207 | CR fitness (12 min walking test), higher is better | Clinician assessment | Ob | MSK | Exercise |
| McNeely, M | | 2010 | | 6 | 166 | 158 | 324 | Shoulder flexion ROM in degrees | Clinician assessment | Ob | MSK | Exercise |
| Main, E | | 2010 | | 7 |  |  | 201 | Pulmonary function (FEV1) | Clinician assessment | Ob | CR | Chest PT |
| Davies, E | | 2010 | | 9 | 1554 | 1555 | 3109 | Health-related QoL | Self-report | Sub | CR | Exercise |
| Busch, A | | 2008 | | 6 | 188 | 161 | 349 | Tender points number | Clinician assessment (self-reported pain at examination] | Sub | MSK | Exercise |
| Liu, C | | 2009 | | 33 | 1076 | 1096 | 2172 | Main function measure (higher is better) | Self-report (clinician assessment] | Sub | MSK | Exercise |
| Furlan, A | 2011 | | 3 | | 188 | 168 | 356 | Pain (higher is worse) | Self-report | Sub | MSK | Acupuncture |
| Fransen, M | 2009 | | 5 | | 102 | 102 | 204 | Pain (higher is worse) | Self-report | Sub | MSK | Exercise |
| Ostelo, R | 2011 | | 3 | | 77 | 45 | 122 | Pain (higher is worse) | Self-report | Sub | MSK | Exercise |
| Taylor, R | 2010 | | 12 | | 817 | 740 | 1557 | Exercise capacity 3-12 mo (higher is better) | Clinician assessment | Ob | CR | Exercise |
| Harvey, L | 2010 | | 8 | | 193 | 186 | 379 | Active knee flexion (ROM) | Clinician assessment | Ob | MSK | Exercise |
| Mead, GE | 2010 | | 23 | | 476 | 431 | 907 | Reduction of depression symptoms post-tx (lower is better) | Clinician assessment | Sub | Neuro | Exercise |
| Edmonds, M | 2010 | | 5 | | 143 | 143 | 286 | Chalder fatigue scale (higher is worse) | Self-report | Sub | MSK, education | Exercise |
| Howe, TE | 2008 | | 5 | | 140 | 164 | 304 | Gait speed (20 min walk test) (s), (lower is better) | Clinician assessment | Ob | MSK | Exercise |
| Fransen, M | 2009 | | 31 | | 2074 | 1542 | 3616 | Pain (higher is worse) | Self-Report | Sub | MSK | Exercise |
| Lin, CH | 2008 | | 8 | |  |  | 513 | Activity limitation questionnaire (activity level) higher is better | Self-Report/performance test | Sub | MSK | Exercise; other |
| Rutjes, AW | 2010 | | 5 | | 177 | 143 | 320 | Pain (higher is worse) | Self-Report | Sub | MSK | Exercise, other |
| Woodford, HJ | 2009 | | 5 | | 47 | 44 | 91 | Change in ROM (higher is better) | Clinician Assessment | Ob | Neuro | PA, exercise |
| Saunders, DH | 2009 | | 7 | | 194 | 177 | 371 | Gait speed (m/min) | Clinician Assessment | Ob | MSK | Exercise |
| O'Brien , K | 2010 | | 5 | | 145 | 131 | 276 | Vo2 max (ml/kg/min) | Clinician Assessment | Ob | CR | Exercise |
| Sirtoti, V | 2009 | | 6 | | 96 | 88 | 184 | Disability post-intervention. (higher is better] | Clinician assessment | Sub | Neuro | Other |
| Hayden, J | 2011 | | 13 | | 704 | 669 | 1373 | Function measure (Oswestry low back pain disability, lower is better) | Self-report | Sub | MSK | Exercise, other |
| Orozco, LJ | 2008 | | 6 | | 1668 | 1647 | 3315 | Change in fasting plasma glucose (mg/dl) | Laboratory measure (physiological measures) | Ob | Other | Exercise, education |
| De Morton, N | 2009 | | 5 | | 1621 | 1857 | 3478 | Acute hospital length of stay | Administrative data | Ob | MSK | Other |
| Mehrholz, J | 2010 | | 7 | | 76 | 77 | 153 | Gait speed | Clinician assessment | Ob | Neuro | Exercise |
| Shaw, K | 2009 | | 15 | | 595 | 484 | 1079 | Wt change in kilograms (higher is worse) | Clinician assessment | Ob | CR | Exercise |
| Handholl, H | 2009 | | 8 | | 817 | 846 | 1663 | Length of hospital stay | Administrative data | Ob | MSK | Other |
| Effing, T | 2009 | | 6 | | 381 | 317 | 698 | HRQOL: SGRQ total (0-100, lower is better) | Self-report | Sub | CR education, prevention | Education |
| Bendermacher, B | 2009 | | 6 | | 118 | 118 | 236 | Maximal treadmill walking distance | Clinician assessment | Ob | Other (cardiovascular) | Exercise |
| Bonaiuti D, | 2009 | | 8 | | 166 | 170 | 336 | BMD | Clinician assessment | Ob | MSK | Exercise |
| Foster, C | 2009 | | 17 | | 4395 | 3203 | 7598 | Change in self-reported physical activity between BL and follow-up | Self-report | Sub | Other (GYN) | Other |
| Jolliffe, J | 2009 | | 8 | | 610 | 588 | 1198 | Total cholesterol | Lab measure (physiological measures) | Ob | CR | Exercise |
| Katalinic, O | 2010 | | 7 | | 96 | 97 | 193 | Joint mobility (ROM) | Clinician assessment | Ob | MSK | Manual Therapy, exercise |
| Puhan, M | 2010 | | 5 | |  |  | 279 | CRQ (higher is better] | Self-report | Ob | CR | Exercise |
| Kramer, M | 2010 | | 6 | | 280 | 276 | 556 | Birth weight (higher is better] | Clinician assessment | Ob | Other | Exercise |
| Rutjes, AW | 2010 | | 11 | | 275 | 190 | 465 | Pain (higher is worse) | Self-report | Sub | MSK | PA |
| Watson, L | 2008 | | 6 | | 129 | 112 | 241 | Treadmill walking distance | Clinician assessment | Ob | Other | Exercise |
| Manheimer E, | 2010 | | 7 | | 902 | 871 | 1773 | Pain (higher is worse) | Self-report | Sub | MSK | Acupuncture, exercise |

BL = baseline; BMD = bone mineral density; CR = cardiorespiratory; CRQ = Chronic respiratory disease questionnaire; FEV1 =Force Expiratory Volume ; GYN = gynaecological; Lab = laboratory; min = minute; mo = month(s); MSK = musculoskeletal; Neuro = neurology; Ob = objective; PA = physical agents; PT = physiotherapy; ROM = range of motion; Sub = subjective; tx = treatment; wt = weight ; Pt=patients
