# Supplementary material for: PEDro or Cochrane to Assess the Quality of Clinical Trials? A Meta-Epidemiological Study
Source: PLoS One. 2015 Jul 10;10(7):e0132634. doi: 10.1371/journal.pone.0132634 (PMC4498768; doi:10.1371/journal.pone.0132634)
Supplement: S3 Table — (DOC) [file pone.0132634.s004.doc]

**Table S3. Identity of trials of adequate quality based on different PeDro cutoffs, Cochrane approach, and results from the original Cochrane review.**

| **Author** | **PEDro >5** | **PEDro >6** | **PEDro>7** | **PEDro>8** | **Cochrane Domain Approach** | **Original Cochrane Review** | **Quality evaluation in MA** |
| --- | --- | --- | --- | --- | --- | --- | --- |
| Pollock, A | Duncan P, 1998  Green J, 2002  Wade D, 1992  Duncan P, 2003 | Duncan P, 1998  Green J, 2002  Wade D, 1992  Duncan P, 2003 | Duncan P, 1998  Green J, 2002  Duncan P, 2003 | Duncan P, 1998  Green J, 2002 | Green J, 2002 | Duncan P, 1998  Green J, 2002  Wade D, 1992  Duncan P, 2003  Ozdemir , 2001 | Included RCTs and qRCTs with or without blinding of pts, therapists, and assessors regardless of quality; RoB tool used |
| States, R | Green J, 2002  Wade D, 1992  Dean C, 2000  Salbach N, 2004  Yang 2006  Yang 2007 | Green J, 2002  Wade D, 1992  Salbach N, 2004  Yang 2006  Yang 2007 | Green J, 2002  Salbach N, 2004  Yang 2006  Yang 2007 | Green J, 2002  Salbach N, 2004 | Wade D, 1992  Salbach N, 2004  Yang 2007 | Green J, 2002  Wade D, 1992  Dean C, 2000  Salbach N, 2004  Yang 2006  Yang 2007 | Included RCTs regardless of quality; PEDro scale and Cochrane Handbook used |
| Schaafsma, F | Bendix A, 1996  Corey D, 1996  Jensen I, 2001  Mitchell R, 1994  Skouen J, 2002 | Bendix A, 1996  Jensen I, 2001 | Jensen I, 2001 | No high quality trials | Corey D, 1996  Jensen I, 2001  Skouen J, 2002 | Bendix A, 1996  Corey D, 1996  Jensen I, 2001  Mitchell R, 1994  Skouen J, 2002 | Included RCTs or cluster RCTs regardless of quality; RoB tool used |
| Markes, M | Campbell A, 2005  Segal R, 2001 | No high quality trials | No high quality trials | No high quality trials | No high quality trials | Campbell A, 2005  Segal R, 2001  Mock V, 1997  Drouin J, 2002 | Included RCTs and qRCTs regardless of quality; van Tulder criteria used with 2 deviations- omitted blinding of pts and care providers, included blinding of outcome assessment |
| McNeely, M | Beurskens C, 2007  Cinar N, 2008  Wingate, 1989 | Beurskens C, 2007  Wingate, 1989 | Beurskens C, 2007 | No high quality trials | No high quality trials | Beurskens C, 2007  Box 2002  Cinar N, 2008  Ferreira De Rezende, 2006  Kilgour R, 2008  Wingate, 1989 | Included RCTs regardless of quality; 6-point scale used that included allocation concealment and modified Jadad 5-point scale |

MA = meta-analysis; PEDro = Physiotherapy Evidence Database pt = patient(s); qRCT = quasi-randomized controlled trial; RCT = randomized controlled trial; RoB = Risk of Bias; tx = treatment

**Supplementary Table 2 (con’t**)

| **MA** | **PEDro >5** | **PEDro >6** | **PEDro>7** | **PEDro>8** | **Cochrane** | **Original Cochrane Review** | **Quality evaluation in MA** |
| --- | --- | --- | --- | --- | --- | --- | --- |
| Main, E | McIwaine M, 1997 | McIwaine M, 1997 | No high quality trials | No high quality trials | No high quality trials | Darbee J, 1990  Davidson A, 1992  Gaskin L, 1998  McIwaine P, 1991  McIwaine P, 1997  Tyrell JC, 1986  Van Asperen P, 1987 | Included RCTs and qRCTs regardless of quality; Jadad scale used |
| Davies, E | Austin J, 2005  Dracup K, 2007  McKelvie R, 2002  Willenheimer R, 2000  Belardinelli R, 1999  Flynn K, 2009  Koukouvou G, 2004  Passino C, 2006 | Austin J, 2005  Dracup K, 2007  McKelvie R, 2002  Willenheimer R, 2000  Flynn K, 2009 | Austin J, 2005  Dracup K, 2007  McKelvie R, 2002 | Austin J, 2005 | McKelvie R, 2002 | Austin J, 2005  Dracup K, 2007  McKelvie R, 2002  Willenheimer R, 2000  Belardinelli R, 1999  Flynn K, 2009  Klocek, 2005  Koukouvou G, 2004  Passino C, 2006 | Included RCTs regardless of quality; RoB tool used |
| Busch, A | Buckelew S, 1998  Gowans S, 2001  King S, 2002  Schachter C, 2003  Wigers H, 1996 | Buckelew S, 1998  Gowans S, 2001  King S, 2002  Wigers H, 1996 | Gowans S, 2001 | No high quality trials | Schachter C, 2003 | Buckelew S, 1998  Gowans S, 2001  King S, 2002  Schachter C, 2003  Sencan S, 2004  Wigers H, 1996 | Included RCTs regardless of quality; van Tulder and Jadad scale used |

con’t = continued; MA = meta-analysis; PEDro = Physiotherapy Evidence Database; qRCT = quasi-randomized controlled trial; RCT = randomized controlled trial

**Supplementary Table 2 (con’t**)

| **MA** | **PEDro >5** | **PEDro >6** | **PEDro>7** | **PEDro>8** | **Cochrane** | **Original Cochrane Review** | **Quality evaluation in MA** |
| --- | --- | --- | --- | --- | --- | --- | --- |
| Liu, C | Segal R, 2003  Baker K, 2001  Bean J, 2004  Buchner D, 1997  Chandler J, 1998  Chin P, 2006  Damush T, 1999  De Vreede P, 2007  Donald I, 2000  Foley A, 2003  Hiatt W, 1994  Jette A, 1999  Katznelson L, 2006  Latham N, 2003  Liu-Ambroise T, 2005  Mangione K, 2005  Mikesky A, 2006  Miller M, 2006  Moreland J, 2001  Ouellete M, 2004  Sims J, 2006  Singh N, 1997  Topp R, 2002  Tracy B, 2004  Tsutsumi, 1997  Westhoff M, 2000  Singh N, 1997 | Segal R, 2003  Baker K, 2001  Bean J, 2004  Buchner D, 1997  Chandler J, 1998  Chin P, 2006  Foley A, 2003  Jette A, 1999  Katznelson L, 2006  Latham N, 2003  Liu-Ambroise T, 2005  Miller M, 2006  Moreland J, 2001  Ouellete M, 2004  Sims J, 2006  Singh N, 1997  Singh N, 2005  Etiinger W, 1997 | Segal R, 2003  Baker K, 2001  Buchner D, 1997  Foley A, 2003  Latham N, 2003  Miller M, 2006  Moreland J, 2001  Ouellete M, 2004  Singh N, 2005 | Segal R, 2003  Foley A, 2003  Latham N, 2003  Miller M, 2006  Moreland J, 2001 | Baker, K | Segal R, 2003  Baker K, 2001  Bean J, 2004  Boshuizen H, 2005  Buchner D, 1997  Chandler J, 1998  Chin P, 2006  Damush T, 1999  De Vreede P, 2007  Donald I, 2000  Foley A, 2003  Hiatt W, 1994  Jette A, 1999  Katznelson L, 2006  Latham N, 2003  Liu-Ambroise T, 2005  Mangione K, 2005  Mikesky A, 2006  Miller M, 2006  Misko T, 2003  Moreland J, 2001  Ouellete M, 2004  Schilke J, 1996  Seyness O, 2004  Sims J, 2006  Singh N, 1997  Topp R, 2002 | Included RCTs regardless of quality; Cochrane Bone, Joint and Muscle Trauma Groups’ former evaluation tool used |

con’t = continued; MA = meta-analysis; PEDro = Physiotherapy Evidence Database; RCT = randomized controlled trial

**Supplementary Table 2 (con’t)**

| **MA** | **PEDro >5** | **PEDro >6** | **PEDro>7** | **PEDro>8** | **Cochrane** | **Original Cochrane Review** | **Quality evaluation in MA** |
| --- | --- | --- | --- | --- | --- | --- | --- |
| Liu, C con’t | Etiinger W, 1997  Singh N, 2005 |  |  |  |  | Tracy B, 2004  Tsutsumi, 1997  Westhoff M, 2000  Singh N, 1997  Etiinger W, 1997  Singh N, 2005 |  |
| Furlan, A | Cherkin, D  Giles L, 2003 | Cherkin, D  Giles L, 2003 | No high quality trials | No high quality trials | Giles L, 1999 | Cherkin D,  Giles L, 2003  Giles L, 1999 | Included RCTs, excluded 2 studies with fatal quality flaws; criteria from updated method guidelines for systematic reviews in the Cochrane Back Review Group used |
| Fransen, M | Foley A, 2003  Fransen M, 2007  Hopmann-Rock M, 2000  Tak E, 2005  Van Baar, 1998 | Foley A, 2003  Fransen M, 2007  Hopmann-Rock M, 2000  Tak E, 2005  Van Baar, 1998 | Foley A, 2003  Fransen M, 2007  Tak E, 2005  Van Baar, 1998 | Foley A, 2003  Fransen M, 2007  Van Baar, 1998 | Foley A, 2003  Fransen M, 2007  Tak E, 2005  Van Baar, 1998 | Foley A, 2003  Fransen M, 2007  Hopmann-Rock M, 2000  Tak E, 2005  Van Baar, 1998 | Included RCTs and qRCTs regardless of quality; Cochrane recommended methods and Jadad scale used |
| Ostelo, R | Dolan P, 2000  Filiz M, 2005 | Dolan P, 2000  Filiz M, 2005 | No high quality trials | No high quality trials | No high quality trials | Dolan P, 2000  Filiz M, 2005  Yilmaz F, 2003 | Included RCTs regardless of quality; criteria from the Cochrane Back Review Group and GRADE approach used |
| Taylor, R | Arthur H, 2002  Dalal H, 2007  Gordon N, 2002  Jolly K, 2007  Marchionni N, 2007  Wu S, 2006 | Dalal H, 2007  Jolly K, 2007  Wu S, 2006 | Jolly K, 2007 | Jolly K, 2007 | Arthur H, 2002  Dalal H, 2007  Jolly K, 2007  Wu S, 2006 | Arthur H, 2002  Carlson J, 2000  Dalal H, 2007  Daskapan A, 2005  Gordon N, 2002  Jolly K, 2007 | Included RCTs and qRCTs regardless of quality; RoB tool used |

con’t = continued; MA = meta-analysis; PEDro = Physiotherapy Evidence Database; qRCT = quasi-randomized controlled trial; RCT = randomized controlled trial; RoB = Risk of Bias

**Supplementary Table 2 (con’t)**

| **MA** | **PEDro >5** | **PEDro >6** | **PEDro>7** | **PeDro>8** | **Cochrane** | **Original Cochrane Review** | **Quality evaluation in MA** |
| --- | --- | --- | --- | --- | --- | --- | --- |
| Taylor, R con’t |  |  |  |  |  | Kassaian M, 2000  Marchionni N, 2007  Miller N, 1984  Wu S, 2006  Sparks B, 1993  Bell J, 1998 |  |
| Harvey, L | Chiarello C, 1997  Denis M, 2006  Huang D, 2003  May L, 1999  McInnes J, 1992  Lenssen T, 2008 | Denis M, 2006  Lenssen T, 2008  McInnes J, 1992 | Denis M, 2006  Lenssen T, 2008 | Denis M, 2006  Lenssen T, 2008 | Denis M, 2006  Lenssen T, 2008  May L, 1999 | Chiarello C, 1997  Denis M, 2006  Huang D, 2003  Lau K, 2001  Lenssen T, 2008  May L, 1999  McInnes J, 1992  Ng T, 1999 | Included RCTs regardless of quality; GRADE approach used |
| Mead, GE | Singh N, 2005  Blumenthal J, 1999  Chou K, 2004  Dunn A, 2002  Doyne E, 1987  Klein M, 1985  Knubben K, 2007  Mather A, 2001  Martinsen E, 1985  McNeil J, 1991  Singh N, 1997  Tsang H, 2006  Blumenthal J, 2007 | Singh N, 2005  Blumenthal J, 1999  Dunn A, 2002  Knubben K, 2007  Mather A, 2001  Singh N, 1997  Blumenthal J, 2007 | Singh N, 2005  Blumenthal J, 2007  Dunn A, 2002  Knubben K, 2007  Mather A, 2001 | Blumenthal J, 2007  Dunn A, 2002  Mather A, 2001 | Singh N, 2005  Blumenthal J, 2007  Mather A, 2001 | Singh N, 2005  Blumenthal J, 1999  Bonnet L, 2005  Chou K, 2004  Dunn A, 2002  Doyne E, 1987  Epstein D, 1986  Fremont J, 1987  Klein M, 1985  Knubben K, 2007  Mather A, 2001  Martinsen E, 1985  McNeil J, 1991 | Included RCTs regardless of quality; assessed for allocation concealment, ITT, and blinding of outcome assessor |

con’t = continued; GRADE = Grading of Recommendations Assessment, Development and Evaluation; ITT = intention to treat; MA = meta-analysis; PEDro = Physiotherapy Evidence Database; RCT = randomized controlled trial;

**Supplementary Table 2 (con’t)**

| **MA** | **PEDro >5** | **PEDro >6** | **PEDro>7** | **PEDro>8** | **Cochrane** | **Original Cochrane Review** | **Quality evaluation in MA** |
| --- | --- | --- | --- | --- | --- | --- | --- |
| Mead, GE |  |  |  |  |  | Mutrie N, 1988  Nabkasorn C, 2005  Orth D, 1979  Setaro J, 1985  Singh N, 1997  Reuter M, 1984  Tsang H, 2006  Veale, 1992  Blumenthal J, 2007  Hess-Homeier, 1981 |  |
| Edmonds, M | Wearden A (Appleby, 1995)  Fulchner K, 1997  Moss-Morris R, 2003  Powell P, 2001  Wallman K, 2004 | Fulchner K, 1997  Moss-Morris R, 2003  Powell P, 2001 | Fulchner K, 1997  Moss-Morris R, 2003  Powell P, 2001 | Moss-Morris R, 2003 | No high quality trials | Wearden A (Appleby, 1995)  Fulchner K, 1997  Moss-Morris R, 2003  Powell P, 2001  Wallman K, 2004 | Included RCTs regardless of quality; criteria from the Cochrane Collaboration Handbook and the CCDAN Quality Rating System used |
| Howe, TE | Buchner D, 1997  Wolfson L, 1996  Krebs D, 1998 | Buchner D, 1997  Wolfson L, 1996  Krebs D, 1998 | Buchner D, 1997 | No high quality trials | Wolfson L, 1996 | Boshuizen H, 2005  Buchner D, 1997  Cress M, 1999  Wolfson L, 1996  Krebs D, 1998 | Included RCTs and qRCTs regardless of quality; modified Cochrane Bone Joint & Muscle Trauma Group’s quality assessment tool, Delphi and Maastricht-Amsterdam used |
| Fransen, M | Baker K, 2001  Foley A, 2003  Mikesky A, 2006  Topp R, 2002  Fransen M, 2007  Hopmann-Rock M, 2000  Van Baar, 1998 | Baker K, 2001  Foley A, 2003  Fransen M, 2001  Hopmann-Rock M, 2000  Van Baar, 1998  Bennell K, 2005  Deyle G, 2000 | Baker K, 2001  Foley A, 2003  Fransen M, 2007  Van Baar, 1998  Bennell K, 2005  Deyle G, 2000 | Foley A, 2003  Fransen M, 2007  Van Baar, 1998  Bennell K, 2005  Hay E, 2006  Messier S, 2004  Quilty B, 2003 | Foley A, 2003  Fransen M, 2007  Van Baar, 1998  Bennell K, 2005  Ettinger W, 1997  Messier S, 2004  Thomas K, 2002 | Baker K, 2001  Foley A, 2003  Mikesky A, 2006  Topp R, 2002  Fransen M, 2007  Hopmann-Rock M, 2000  Van Baar, 1998 | Included RCTs and qRCTs regardless of quality; Jadad scale supplemented with an evaluation of the reported methods for allocation concealment used, as well as a modified system for grading the strength of evidence |

CCDAN = Cochrane Collaboration Depression, Anxiety and Neurosis Review Group; con’t = continued; MA = meta-analysis; PEDro = Physiotherapy Evidence Database; qRCT = quasi-randomized controlled trial; RCT = randomized controlled trial

**Supplementary Table 2 (con’t)**

| **MA** | **PEDro >5** | **PEDro >6** | **PEDro>7** | **PEDro>8** | **Cochrane** | **Original Cochrane Review** | **Quality evaluation in MA** |
| --- | --- | --- | --- | --- | --- | --- | --- |
| Fransen, M con’t | Bautch J, 1997  Bennell K, 2005  Deyle G, 2000  Ettinger W, 1997  Fransen M, 2001  Fransen M, 2007  Gur H, 2002  Hay E, 2006  Huang M, 2003  Kovar P, 1992  Maurer B, 1999  Messier S, 2004  O'Rheilly S, 1999  Peloquin, 1999  Thorstensson C, 2005  Thomas K, 2002  Talbot L, 2003  Song R, 2003  Rogind H, 1998  Quilty B, 2003  Huang M, 2005 | Ettinger W, 1997  Fransen M, 2001  Fransen M, 2001  Hay E, 2006  Huang M, 2003  Maurer B, 1999  Messier S, 2004  O'Rheilly S, 1999  Peloquin, 1999  Thorstensson C, 2005  Thomas K, 2002  Rogind H, 1998  Quilty B, 2003  Huang M, 2005 | Hay E, 2006  Huang M, 2005  Messier S, 2004  O'Rheilly S, 1999  Thomas K, 2002  Rogind H, 1998  Quilty B, 2003 |  | Song R, 2003  Rogind H, 1998  Quilty B, 2003 | Bautch J, 1997  Bennell K, 2005  Deyle G, 2000  Ettinger W, 1997  Fransen M, 2001  Gur H, 2002  Hay E, 2006  Huang M, 2005  Hughes S, 2004  Keefe F, 2004  Kovar P, 1992  Maurer B, 1999  Messier S, 2004  Minor M, 1989  O'Rheilly S, 1999  Peloquin, 1999  Thorstensson C, 2005  Thomas K, 2002  Talbot L, 2003  Song R, 2003  Rogind H, 1998  Quilty B, 2003  Huang M, 2003  Schilke, 1996 |  |
|  |
| Lin, CH | Egol K, 2000  Hedstrom M, 1994  Lethonen H, 2003 | Lethonen H, 2003 | No high quality trials | No high quality trials | Egol K, 2000  Lethonen H, 2003  Rasmussen S, 2000 | Egol K, 2000  Finsen V, 1989  Hedstrom M, 1994  Lethonen H, 2003  Tropp H, 1995  Vioreanu M, 2007  Rasmussen S, 2000 | Included RCTs and qRCTs regardless of quality; PEDro scale used |

con’t = continued; MA = meta-analysis; PEDro = Physiotherapy Evidence Database; qRCT = quasi-randomized controlled trial; RCT = randomized controlled trial;

**Supplementary Table 2 (con’t)**

| **MA** | **PEDro >5** | | **PEDro >6** | **PEDro>7** | **PEDro>8** | **Cochrane** | **Original Cochrane Review** | **Quality evaluation in MA** |
| --- | --- | --- | --- | --- | --- | --- | --- | --- |
| Rutjes, AW | | Huang M, 2005  Cetin N, 2008  Falconer J, 1992  Huang M, 2005  Ozgonenel L, 2009 | Huang M, 2005  Cetin N, 2008  Falconer J, 1992  Ozgonenel L, 2009 | Huang M, 2005  Falconer J, 1992  Ozgonenel L, 2009 | No high quality trials | No high quality trials | Huang M, 2005  Cetin N, 2008  Falconer J, 1992  Huang M, 2005  Ozgonenel L, 2009 | Included RCTs and qRCTs regardless of quality; assessed randomization, blinding and adequacy of analyses, as well as GRADE for the overall body of evidence |
| Woodford, HJ | | Binder S, 1981  Burnside I, 1982  Cozean C, 1988 | Burnside I, 1982  Cozean C, 1988 | Burnside I, 1982 | No high quality trials | No high quality trials | Binder S, 1981  Burnside I, 1982  Bradley L, 1998  Cozean C, 1988  Lee S, 1985 | Included RCTs and qRCTs regardless of quality; criteria from the Cochrane Collaboration’s grading system used for allocation concealment |
| Saunders, DH | | Bateman A, 2001  Eich H, 2004  Pohl M, 2002  Salbach N, 2004  Pohl M, 2007 | Bateman A, 2001  Eich H, 2004  Pohl M, 2002  Salbach N, 2004  Pohl M, 2007 | Bateman A, 2001  Eich H, 2004  Pohl M, 2007  Salbach N, 2004 | Eich H, 2004  Pohl M, 2007 | No high quality trials | Bateman A, 2001  Da Cunha I, 2002  Eich H, 2004  Glasser L, 1986  Pohl M, 2002  Salbach N, 2004  Pohl M, 2007 | Included RCTs that were single-blinded or open regardless of quality; assessed randomization, allocation concealment, blinding and ITT |
| O'Brien , K | | Baigis J, 2002  Mutimura E, 2008  Smith B, 2001 | No high quality trials | No high quality trials | No high quality trials | No high quality trials | Baigis J, 2002  Mutimura E, 2008  Perna F, 1999  Smith B, 2001  Stringer W, 1998 | Included RCTs regardless of quality; Jadad scale used |
| Sirtoti, V | | Lin K, 2007  Ploughman M, 2004  Wu C, 2007  Dahl A, 2008  Myint J, 2008  Wu C, 2007 | Lin K, 2007  Wu C, 2007  Wu C, 2007  Dahl A, 2008  Myint J, 2008 | Wu C, 2007  Dahl A, 2008  Myint J, 2008 | Dahl A, 2008 | Wu C, 2007  Dahl A, 2008 | Lin K, 2007  Ploughman M, 2004  Wu C, 2007  Dahl A, 2008  Myint J, 2008  Wu C, 2007 | Included RCTs and qRCTs regardless of quality; criteria from the Cochrane Handbook for Systematic Reviews of Interventions used |

con’t = continued; GRADE = Grading of Recommendations Assessment, Development and Evaluation; ITT = intention to treat; MA = meta-analysis; PEDro = Physiotherapy Evidence Database; qRCT = quasi-randomized controlled trial; RCT = randomized controlled trial;

**Supplementary Table 2 (con’t)**

| **MA** | **PEDro >5** | | **PEDro >6** | **PEDro>7** | **PEDro>8** | **Cochrane** | **Original Cochrane Review** | **Quality evaluation in MA** |
| --- | --- | --- | --- | --- | --- | --- | --- | --- |
| Hayden, J | | Preyde M, 2000  Turner J, 1990  Bronfort G, 1996  Deyo R, 1990  Frost H, 1995  Gur A, 2003  Kankaanpaa M, 1999  Niemisto L, 2003  Rasmussen-Barr E, 2003  Torstensen T, 1998  Yelland M, 2004  Bendix A, 1995  Frost H, 2004 | Preyde M, 2000  Bronfort G, 1996  Deyo R, 1990  Frost H, 1995  Frost H, 2004  Niemisto L, 2003  Torstensen T, 1998  Yelland M, 2004 | Bronfort G, 1996  Frost H, 1995  Niemisto L, 2003  Yelland M, 2004 | Niemisto L, 2003 | No high quality trials | Preyde M, 2000  Turner J, 1990  Bronfort G, 1996  Deyo R, 1990  Frost H, 1995  Gur A, 2003  Kankaanpaa M, 1999  Niemisto L, 2003  Rasmussen-Barr E, 2003  Torstensen T, 1998  Yelland M, 2004  Bendix A, 1995  Frost H, 2004 | Included RCTs regardless of quality; Jadad scale used including randomization, allocation concealment, adequacy of follow-up and blinding of outcome assessor |
| Orozco, LJ | | Bo S, 2007  Knowler W, 2002  Tuomilehto J, 2001  Ramachandran A, 2006  Oldroyd J, 2005  Wing R, 1998 | Bo S, 2007  Knowler W, 2002  Tuomilehto J, 2001  Oldroyd J, 2005  Wing R, 1998 | Bo S, 2007 | No high quality trials | Bo S, 2007  Knowler W, 2002 | Bo S, 2007  Knowler W, 2002  Tuomilehto J, 2001  Ramachandran A, 2006  Oldroyd J, 2005  Wing R, 1998 | Included RCTs regardless of quality; RoB tool used |
| De Morton, N | | Asplund K, 2000  Counsell S, 2000 | Asplund K, 2000  Counsell S, 2000 | Counsell S, 2000 | No high quality trials | No high quality trials | Landefeld C, 1995  Slaets J, 1997  Asplund K, 2000  Counsell S, 2000  Collard A, 1985 | Included prospective RCTs and CCTs regardless of quality; PEDro scale used |

con’t = continued; CCT = clinical controlled trial; MA = meta-analysis; PEDro = Physiotherapy Evidence Database; RCT = randomized controlled trial;

**Supplementary Table 2 (con’t)**

| **MA** | **PEDro >5** | | **PEDro >6** | **PEDro>7** | **PEDro>8** | **Cochrane** | **Original Cochrane Review** | **Quality evaluation in MA** |
| --- | --- | --- | --- | --- | --- | --- | --- | --- |
| Mehrholz, J | | Miyai I, 2000 (1999)  Pohl M, 2003  Cakit B, 2007  Fisher B, 2008  Kurtais Y, 2008  Protas E, 2005 | Pohl M, 2003  Kurtais Y, 2008 | No high quality trials | No high quality trials | Pohl M, 2003  Kurtais Y, 2008 | Miyai I, 2000 (1999)  Pohl M, 2003  Cakit B, 2007  Fisher B, 2008  Kurtais Y, 2008  Protas E, 2005  Miyai I, 2002 | Included RCTs and randomized controlled cross-over trials regardless of quality; PEDro scale used |
| Shaw, K | | Svendsen O, 1993  Gordon N, 1997  Hays N, 2004  Janssen I, 2002  Ross R, 1996  Thong F, 2000  Van Aggel-Leijssen, 2001  Whatley J, 1994  Wing R, 1998 | Svendsen O, 1993  Ross R, 1996  Wing R, 1998 | No high quality trials | No high quality trials | No high quality trials | Nieman D, 1998  Wood P, 1991  Svendsen O, 1993  Neumark-Sztainer D, 1995  Gordon N, 1997  Hays N, 2004  Janssen I, 2002  Kiernan M, 2001  Ross R, 1996  Stefanick M, 1998  Thong F, 2000  Van Aggel-Leijssen, 2001  Wadden T, 1997  Whatley J, 1994  Wing R, 1998 | Included RCTs regardless of quality; criteria by Schultz and Jadad used |
| Handholl, H | | Gilchrist W, 1988  Stenvall M, 2007  Cameron I, 1993  Kennie D, 1988  Naglie G, 2002  Shyu Y, 2008 | Stenvall M, 2007  Cameron I, 1993  Kennie D, 1988  Naglie G, 2002  Shyu Y, 2008 | Naglie G, 2002  Shyu Y, 2008 | Naglie G, 2002 | Naglie G, 2002 | Galvard H, 1995  Gilchrist W, 1988  Stenvall M, 2007  Day G-Swanson 1998  Cameron I, 1993  Kennie D, 1988  Naglie G, 2002  Shyu Y, 2008 | Included RCTs and qRCTs regardless of quality; RoB tool used |

con’t = continued; MA = meta-analysis; PEDro = Physiotherapy Evidence Database; qRCT = quasi-randomized controlled trial; RCT = randomized controlled trial; RoB = Risk of Bias

**Supplementary Table 2 (con’t)**

| **MA** | **PEDro >5** | | **PEDro >6** | **PEDro>7** | **PEDro>8** | **Cochrane** | **Original Cochrane Review** | **Quality evaluation in MA** | |
| --- | --- | --- | --- | --- | --- | --- | --- | --- | --- |
| Effing, T | | Bourbeau J, 2003  Boxall A, 2005  Coultas D, 2005  Gallefos F, 1999  Monninkhof E, 2003  Watson P, 1997 | Bourbeau J, 2003  Gallefos F, 1999  Monninkhof E, 2003 | Bourbeau J, 2003  Monninkhof E, 2003 | Bourbeau J, 2003 | Bourbeau J, 2003  Coultas D, 2005 | Bourbeau J, 2003  Boxall A, 2005  Coultas D, 2005  Gallefos F, 1999  Monninkhof E, 2003  Watson P, 1997 | Included RCTs and CCTs regardless of quality; Jadad scale used |  |
|  | |
| Bendermacher, B | | Chetham D, 2004  Regensteiner J, 1997 | No high quality trials | No high quality trials | No high quality trials | No high quality trials | Chetham D, 2004  Nielsen SL, 1975  Degisher S, 2002  Patterson R, 1997  Regensteiner J, 1997  Savage P, 2001 | Included RCTs and CCTs regardless of quality; assessed allocation concealment and follow-up based on the Cochrane Collaboration Handbook | |
| Bonaiuti D, | | Nelson M, 1994  Grove K, 1992  Hatori M, 1993  Smidt G, 1992 | Hatori M, 1993 | No high quality trials | No high quality trials | No high quality trials | Lord S, 1996  Nelson M, 1994  Pruitt L, 1996  Grove K, 1992  Hatori M, 1993  Lau E, 1992  Martin & Morris, 1993  Smidt G, 1992 | Included RCTs regardless of quality; Jadad scale used | |

CCT = clinical controlled trial; con’t = continued; MA = meta-analysis; PEDro = Physiotherapy Evidence Database; RCT = randomized controlled trial

**Supplementary Table 2 (con’t)**

| **MA** | **PEDro >5** | | **PEDro >6** | **PEDro>7** | **PEDro>8** | **Cochrane** | **Original Cochrane Review** | **Quality evaluation in MA** |
| --- | --- | --- | --- | --- | --- | --- | --- | --- |
| Foster, C | | Green B, 2002  Smith B, 2000  Calfas K, 2000  Cunningham D, 1987  Elley C, 2003  Goldstein M, 1999  Hillsdon M, 2002  Inoue S, 2003  Marshall A, 2003  Pinto B, 2002  Simons-Morton D, 2001  Stevens W, 1998  Tsuji I, 2000 | Smith B, 2000  Hillsdon M, 2002  Inoue S, 2003  Simons-Morton D, 2001  Tsuji I, 2000 | Simons-Morton D, 2001 | Simons-Morton D, 2001 | No high quality trials | Green B, 2002  King A, 1988  Smith B, 2000  Calfas K, 2000  Cunningham D, 1987  Elley C, 2003  Resnick B, 2002  Goldstein M, 1999  Hillsdon M, 2002  Inoue S, 2003  Kriska A, 1986  Marshall A, 2003  Pinto B, 2002  Simons-Morton D, 2001  Stevens W, 1998  Stewart A, 2001  Tsuji I, 2000 | Included RCTs regardless of quality; assessed randomization, blinding of outcome assessor, baseline differences in physical activity and ITT |
| Jolliffe, J | | Carlsson,  Englom, E  Fletcher, B  Haskell W, 1994  Kallio, V  Ornish, D  Schuler G, 1992 | Haskell W, 1994  Schuler G, 1992 | Haskell W, 1994 | No high quality trials | Haskell W, 1994 | Carlsson R, 1998  Englom E, 1992  Fletcher B, 1994  Haskell W, 1994  Kallio V, 1979  Ornish D, 1990  Schuler G, 1992  Vermeulen A, 1983 | Included RCTs regardless of quality; assessed allocation concealment, losses to follow-up and blind assessment of outcomes |
| Katalinic, O | | Lannin N, 2003  Ada L, 2005  De Jong L, 2006  Dean C, 2000  Gustafsson L, 2006  Lannin N, 2007 | Lannin N, 2003  Ada L, 2005  De Jong L, 2006  Dean C, 2000  Gustafsson L, 2006  Lannin N, 2007 | Lannin N, 2003  Ada L, 2005  De Jong L, 2006  Dean C, 2000  Lannin N, 2007 | Lannin N, 2003  Ada L, 2005  Lannin N, 2007 | Lannin N, 2003  Ada L, 2005  De Jong L, 2006  Dean C, 2000  Lannin N, 2007 | Lai J, 2009  Lannin N, 2003  Ada L, 2005  De Jong L, 2006  Dean C, 2000  Gustafsson L, 2006  Lannin N, 2007 | Included RCTs and CCTs regardless of quality; RoB tool used, as well as GRADE approach |

CCT = controlled clinical trial; con’t = continued; GRADE = Grading of Recommendations Assessment, Development and Evaluation; ITT = intention to treat; MA = meta-analysis; PEDro = Physiotherapy Evidence Database; RCT = randomized controlled trial; RoB = Risk Of Bias

**Supplementary Table 2 (con’t)**

| **MA** | **PEDro >5** | **PEDro >6** | **PEDro>7** | **PEDro>8** | **Cochrane** | **Original Cochrane Review** | **Quality evaluation in MA** |
| --- | --- | --- | --- | --- | --- | --- | --- |
| Puhan, M | Man W, 2004  Carr S, 2009  Eaton T, 2009  Seymor J, 2010 | Eaton T, 2009  Seymor J, 2010 | Eaton T, 2009  Seymor J, 2010 | No high quality trials | No high quality trials | Man W, 2004  Benhke M, 2000  Carr S, 2009  Eaton T, 2009  Seymor J, 2010 | Included RCTs regardless of quality; RoB tool used |
| Kramer, M | Clapp J, 2000 | Clapp J, 2000 | No high quality trials | No high quality trials | No high quality trials | Lee G, 1996  Marquez-Sterling S, 2000  Clapp J, 2000  Collings C, 1983  Erkkola R, 1976  Memari A, 2006 | Included RCTs and qRCTs, one study excluded due to high dropout; RoB tool used |
| Rutjes, AW | Law P, 2004  Cetin N, 2008  Cheing G, 2002  Law P, 2004  Cheing G, 2003 | Law P, 2004  Cetin N, 2008  Law P, 2004 | Law P, 2004  Law P, 2004 | Law P, 2004  Law P, 2004 | No high quality trials | Law P, 2004  Cetin N, 2008  Adedoyin R, 2005  Cheing G, 2002  Bal S, 2007  Fargas-Babjak, 1989  Law P, 2004  Cheing G, 2003  Ng, M  Yurtkuran, M  Grimmer K, 1992 | Included RCTs and qRCTs regardless of quality; RoB tool used but did not assess blinding of therapist or outcome assessor, GRADE approach used for overall body of evidence |
| Watson, L | Collins E, 2005  Hiatt W, 1990  Mika P, 2006  Sanderson B, 2006  Tsai J, 2002 | Mika P, 2006  Sanderson B, 2006’ | No high quality trials | No high quality trials | No high quality trials | Collins E, 2005  Hiatt W, 1990  Mika P, 2006  Sanderson B, 2006  Tsai J, 2002  Hiatt W, 1994 | Included RCTs regardless of quality; assessed allocation concealment as described by Schultz, as well as Jadad scale |

CCT = clinical controlled trial; con’t = continued; GRADE = Grading of Recommendations Assessment, Development and Evaluation; MA = meta-analysis; PEDro = Physiotherapy Evidence Database; qRCT = quasi-randomized controlled trial; RCT = randomized controlled trial; RoB = Risk of Bias

**Supplementary Table 2 (con’t)**

| **MA** | **PEDro >5** | **PEDro >6** | **PEDro>7** | **PEDro>8** | **Cochrane** | **Original Cochrane Review** | **Quality evaluation in MA** |
| --- | --- | --- | --- | --- | --- | --- | --- |
| Manheimer E, | Foster N, 2007  Witt C, 2005  Berman B, 2004  Sangdee C, 2002  Scharf, 2006  Vas J, 2004  Takeda W, 2004 | Foster N, 2007  Witt C, 2005  Berman B, 2004  Sangdee C, 2002  Scharf, 2006  Vas J, 2004  Takeda W, 2004 | Foster N, 2007  Witt C, 2005  Berman B, 2004  Scharf, 2006  Vas J, 2004  Takeda W, 2004 | Foster N, 2007  Witt C, 2005  Scharf, 2006  Vas J, 2004 | Foster N, 2007  Witt C, 2005  Scharf, 2006 | Foster N, 2007  Witt C, 2005  Berman B, 2004  Sangdee C, 2002  Scharf, 2006  Vas J, 2004  Takeda W, 2004 | Included RCTs regardless of quality; RoB tool used, as well as GRADE for the overall body of evidence |

con’t = continued; GRADE = Grading of Recommendations Assessment, Development and Evaluation; MA = meta-analysis; PEDro = Physiotherapy Evidence Database; RCT = randomized controlled trial; RoB = Risk of Bias;
